# The Human Cytomegalovirus UL11 Protein Interacts with the Receptor Tyrosine Phosphatase CD45, Resulting in Functional Paralysis of T Cells

**DOI:** 10.1371/journal.ppat.1002432

**Published:** 2011-12-08

**Authors:** Ildar Gabaev, Lars Steinbrück, Claudia Pokoyski, Andreas Pich, Richard J. Stanton, Reinhard Schwinzer, Thomas F. Schulz, Roland Jacobs, Martin Messerle, Penelope C. Kay-Fedorov

**Affiliations:** 1 Institute of Virology, Hannover Medical School, Hannover, Germany; 2 Department of General, Visceral and Transplantation Surgery, Hannover Medical School, Hannover, Germany; 3 Institute of Toxicology, Hannover Medical School, Hannover, Germany; 4 Section of Medical Microbiology, School of Medicine, Cardiff University, Cardiff, United Kingdom; 5 Department of Clinical Immunology and Rheumatology, Hannover Medical School, Hannover, Germany; Oregon Health and Science University, United States of America

## Abstract

Human cytomegalovirus (CMV) exerts diverse and complex effects on the immune system, not all of which have been attributed to viral genes. Acute CMV infection results in transient restrictions in T cell proliferative ability, which can impair the control of the virus and increase the risk of secondary infections in patients with weakened or immature immune systems. In a search for new immunomodulatory proteins, we investigated the UL11 protein, a member of the CMV RL11 family. This protein family is defined by the RL11 domain, which has homology to immunoglobulin domains and adenoviral immunomodulatory proteins. We show that pUL11 is expressed on the cell surface and induces intercellular interactions with leukocytes. This was demonstrated to be due to the interaction of pUL11 with the receptor tyrosine phosphatase CD45, identified by mass spectrometry analysis of pUL11-associated proteins. CD45 expression is sufficient to mediate the interaction with pUL11 and is required for pUL11 binding to T cells, indicating that pUL11 is a specific CD45 ligand. CD45 has a pivotal function regulating T cell signaling thresholds; in its absence, the Src family kinase Lck is inactive and signaling through the T cell receptor (TCR) is therefore shut off. In the presence of pUL11, several CD45-mediated functions were inhibited. The induction of tyrosine phosphorylation of multiple signaling proteins upon TCR stimulation was reduced and T cell proliferation was impaired. We therefore conclude that pUL11 has immunosuppressive properties, and that disruption of T cell function via inhibition of CD45 is a previously unknown immunomodulatory strategy of CMV.

## Author Summary

The human cytomegalovirus (CMV) belongs to a class of viruses that interferes with the immune response of its host. Accordingly, infection with CMV is a severe risk for immunologically immature newborns and immunocompromised patients such as transplant recipients. The mechanisms by which CMV affects the immune system are not completely understood. Here we show that a CMV protein, pUL11, which is expressed on the surface of cells, binds to leukocytes by interacting with the receptor tyrosine phosphatase CD45. In T cells, CD45 is essential for transmission of activating signals received via the T cell receptor (TCR) to downstream effector molecules that ultimately lead to activation and proliferation of these immune cells. Binding of the CMV pUL11 protein to CD45 on T cells prevents signal transduction via the TCR and restricts T cell proliferation. Interestingly, the mechanism by which the activity of CD45 is regulated is a matter of debate and no specific cellular ligand of CD45 has yet been described. The identification of a first viral ligand for CD45 may provide the means to investigate CD45 regulatory mechanisms and also allow the development of therapies to interfere with CMV-mediated immunomodulation.

## Introduction

Infection of immunocompetent individuals with human cytomegalovirus (CMV) rarely results in symptomatic disease. Following primary infection children and even adults often shed the virus in saliva or urine for weeks or months [1], suggesting that clearance of CMV by the immune system is a complex process. Cellular immunity, in particular Natural Killer (NK) cells and CD8 T cells, has been found to be pivotal in controlling CMV [2], [3]. Yet, despite the induction of strong cellular immune responses and neutralizing antibodies, CMV is able to establish a latent infection, and reactivation as well as reinfection with multiple CMV strains seems to be quite frequent [4]–[6]. These properties of CMV have been ascribed to the expression of a series of viral immunomodulatory proteins [3], [7]. In individuals with weakened or immature immune systems the balance between host immune control and viral immunomodulation can easily be shifted in favor of viral replication, resulting in viremia and end-organ disease associated with morbidity and even mortality in CMV-infected transplant recipients, AIDS patients or children congenitally infected with CMV [8].

It is a long standing observation that T lymphocytes in patients with acute CMV infection display reduced proliferation capacity [9]–[13] that may result in transient immunosuppression associated with an increased risk of secondary infection [14], [15]. A number of mechanisms have been proposed by which CMV may interfere with the priming of T cells as well as with their effector functions. The inhibition of MHC class I antigen presentation pathways by CMV is well established; limiting the recognition and lysis of infected cells by cytolytic T lymphocytes [7], [16]. Another strategy that acts on the ability of T cells to proliferate is the secretion of host and virally encoded suppressive factors from CMV-infected cells; the virus induces enhanced secretion of transforming growth factor β1 and soluble CD83, and itself encodes an interleukin-10 homologue that suppresses T cell proliferation [17]–[20]. Other suppressive functions require direct contact between infected cells and T cells [12]. An example is the upregulation of pro-apoptotic ligands on the surface of CMV-infected dendritic cells that can induce apoptosis in activated T cells [21]. The observation that the fraction of T cells that is not driven into apoptosis is also unable to proliferate normally after contact with CMV-infected cells implies the existence of additional suppressive mechanisms [21]. One possibility could be the interaction of CMV-encoded surface proteins with regulatory or inhibitory receptors on T cells.

Cellular proteins and also immunomodulatory proteins of various viruses that mediate the interaction with surface proteins of immune cells often contain immunoglobulin-like or MHC-like domains [22]–[24]. The CMV genome encodes a number of putative transmembrane proteins with such a property [25], the most prominent being the RL11 family that includes 14 largely uncharacterized proteins. The defining motif of this family is the RL11 domain, which has limited sequence homology to immunoglobulin domains and to the immunomodulatory E3 proteins of adenoviruses [26]. The only member of the RL11 family that has been studied more thoroughly, the TRL11/IRL11 protein, encodes an Fc-receptor that binds human immunoglobulins, presumably mediating escape from recognition by anti-viral immunoglobulins [27], [28]. In this study we focused on another member of the RL11 family, the UL11 protein that has previously been reported to be expressed on the cell surface of CMV-infected cells [29] and therefore has the potential to interact with T cell receptor molecules.

The restricted proliferative capacity of T cells from CMV-infected patients has been linked with defects in T cell receptor (TCR) signaling [30]. There are only a few transmembrane proteins on T cells that may exert negative regulatory effects on TCR signaling, the most prominent being the receptor tyrosine phosphatase CD45. The CD45 protein is an essential regulator of the TCR signaling pathway that determines the sensitivity of T cells to TCR mediated stimulation. The absence of CD45 leads to a severe combined immunodeficiency (SCID) phenotype in humans [31]–[33] and mice [34]–[36]. The key substrate of the CD45 phosphatase in TCR signaling is the Src family kinase Lck [37], which is in close proximity to the TCR and, upon activation by an incoming stimulatory signal, phosphorylates immunoreceptor tyrosine-based activation motifs (ITAMs) in subunits of the CD3-TCR complex [38], [39]. In common with other Src family kinases, Lck is regulated via the phosphorylation status of one inhibitory and one activating tyrosine residue; Y505 and Y394 in Lck [40]. When phosphorylated, the negative regulatory tyrosine, Y505, maintains an intramolecular interaction holding Lck in a closed, inactive conformation [41]–[43]. CD45 dephosphorylates Y505 and releases Lck into an open, primed form, ready to receive a signal from the CD3-TCR complex that results in phosphorylation of Y394 and thereby activates the kinase activity of Lck [44]–[46]. CD45 also has a less favored inhibitory function, to dephosphorylate Y394 [47]–[49]. For TCR signaling to occur, a pool of primed Lck must be available. CD45 is the only phosphatase known to dephosphorylate the inhibitory tyrosine of Lck, and the action of CD45 is therefore essential in setting the threshold at which incoming stimulating signals can be transduced into effects [50].

In this study we show that the CMV UL11 protein interacts with the CD45 receptor phosphatase on the surface of T cells, inhibiting signaling and restricting T cell proliferation.

## Results

### The CMV UL11 ORF encodes a surface expressed glycoprotein

**Figure 1:**
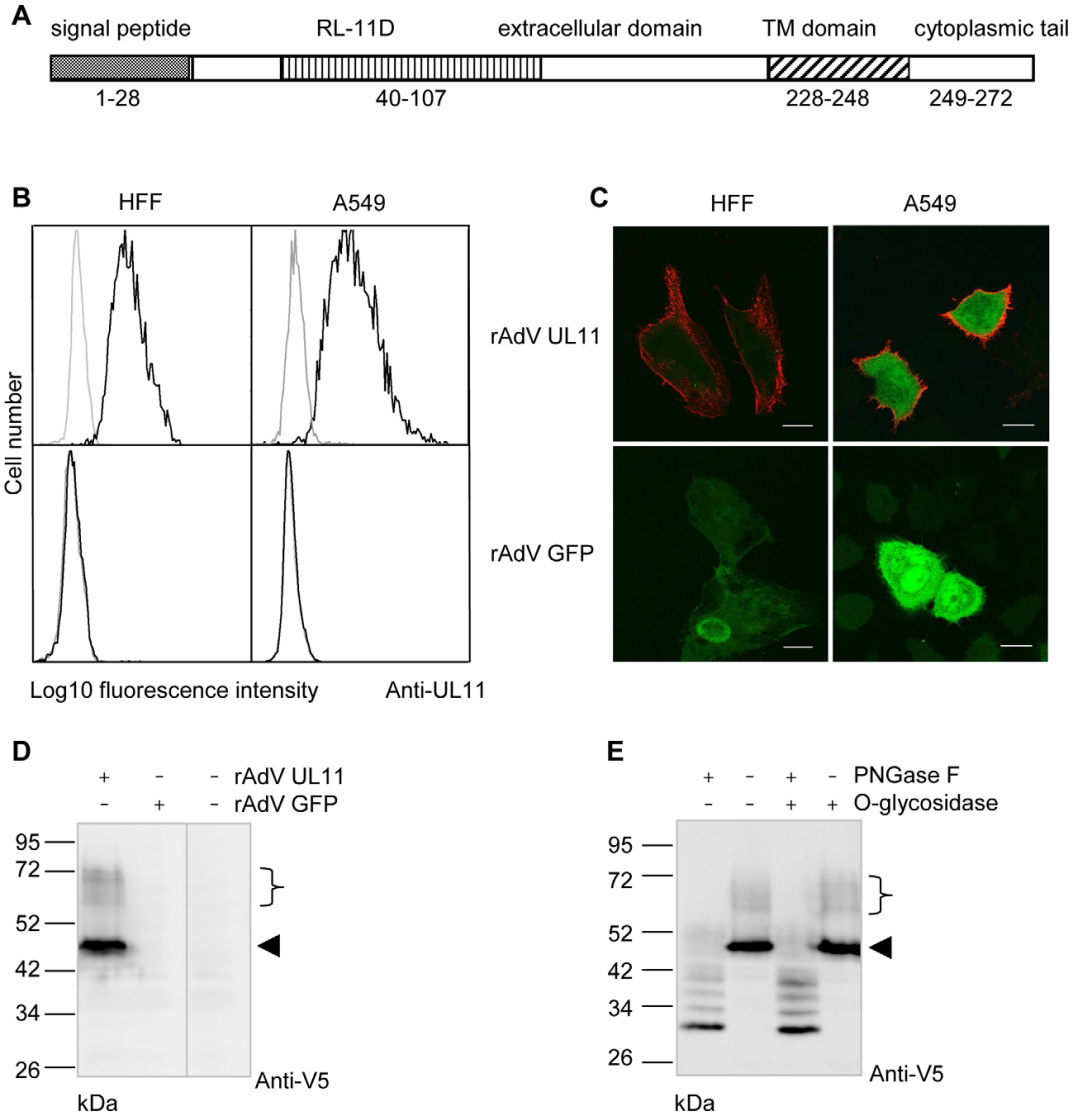
pUL11 is a surface expressed glycoprotein. (A) Predicted structure of the UL11 protein. (B) Flow cytometry with a pUL11-specific rabbit antiserum (black lines) or pre-immune serum (grey lines) of HFF or A549 cells transduced with recombinant adenoviruses expressing UL11 and GFP (rAdV UL11) or GFP alone (rAdV GFP). Gating was on GFP positive cells. (C) Confocal microscopy of HFF and A549 cells transduced as in B) and labeled at the cell surface with the anti-pUL11 serum and an Alexa-568 conjugated anti-rabbit antibody (shown in red). Size bar, 20 µm. (D) Immunoblot with a mouse anti-V5 antibody of lysates of rAdV UL11, rAdV GFP or mock transduced A549 cells. (E) Lysates of rAdV UL11 transduced A549 cells were treated with peptide N-glycosidase F (PNGase F), endo-α-N-acetylgalactosaminidase (O-glycosidase) or untreated as indicated. Immunoblotting was performed as in (D). (D, E) Left margin, molecular mass (in kDa); right margin, the arrowhead and the bracket indicate low and high molecular weight forms of pUL11.

The UL11 protein is predicted to be a type I transmembrane protein (Figure 1A) and has previously been reported to be expressed on the surface of human fibroblasts infected with the highly passaged CMV laboratory strain AD169 [29]. Expression of the protein from more physiologically relevant strains of the virus and in other cell types has not been analyzed, but relatively low levels of UL11 transcription from the Merlin strain of CMV have been observed (Andrew Davison, personal communication). To allow us to work with conveniently detectable levels of pUL11, we therefore used an adenovirus expression system [51] and constructed a recombinant adenovirus (rAdV UL11) expressing pUL11 from the TB40/E strain of CMV [52] and GFP to allow the identification of transduced cells. Using a polyclonal antiserum specific for the predicted N-terminal extracellular domain of UL11 (Figure S1) we detected pUL11 on the surface of A549 lung epithelial cells and human foreskin fibroblasts (HFF) transduced with rAdV UL11, but not with a control adenovirus lacking UL11 (rAdV GFP) by flow cytometry (Figure 1B) and confocal microscopy (Figure 1C). These results confirmed that pUL11 is expressed at the cell surface and furthermore indicated that surface expression of pUL11 does not require the presence of other CMV proteins.

To characterize the UL11 protein, we transduced A549 cells with rAdV UL11 and performed immunoblots of the cell lysates using an antibody specific for the V5 epitope added at the C-terminus of the UL11 protein. A prominent band of 50 kDa and several faint bands with apparent molecular masses ranging from 60 to 72 kDa were detected (Figure 1D). This suggested posttranslational modification of the UL11 protein since the predicted molecular mass of pUL11 is 31 kDa. To investigate potential glycosylation of pUL11, lysates of recombinant adenovirus transduced cells were treated with PNGase F, O-glycosidase, or a combination of the two and immunoblotted (Figure 1E). PNGase F treatment led to a reduction of the molecular mass of pUL11 to approximately the predicted 31 kDa, but treatment with O-glycosidase, either alone or in combination with PNGase F, had no effect. N-linked glycosylation therefore appears to form all or the majority of the post-translational modification of pUL11.

### The extracellular domain of pUL11 interacts with a 200 kDa leukocyte cell surface protein

**Figure 2:**
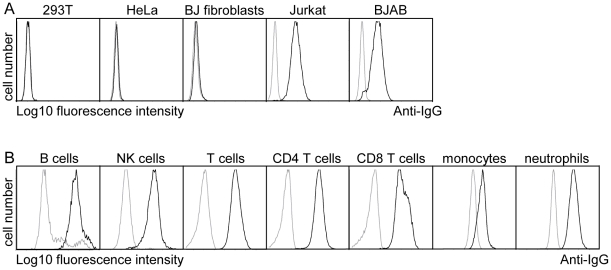
pUL11 interacts with leukocytes. (A) Surface staining of the indicated cell lines with purified UL11Fc (black lines), or the Fc control protein (grey lines). (B) Purified UL11Fc or Fc proteins were incubated with primary PBMCs. Surface markers and cell size were used to set gates for different leukocyte subpopulations.

As pUL11 is expressed on the cell surface, its role could potentially be to interact with proteins on the surface of neighboring cells such as infiltrating immune effector cells in infected tissue. The UL11Fc protein consisting of the pUL11 extracellular domain with the Fc domain of human IgG fused at the C-terminus (Figure S2) was used to measure interactions of pUL11 with five different cell types by flow cytometry. Markedly higher binding of UL11Fc than the control Fc domain (Fc) was detected to the lymphocyte cell lines BJAB and Jurkat but not to the non-hematopoietic cell lines HeLa, 293T or BJ fibroblasts (Figure 2A). Extension of the study to primary PBMCs from a healthy donor indicated interactions of UL11Fc with CD4 and CD8 T cells, B cells, NK cells, monocytes and neutrophils (Figure 2B). These data suggested that pUL11 binds to a protein that is ubiquitously expressed on cells of hematopoietic origin.

**Figure 3:**
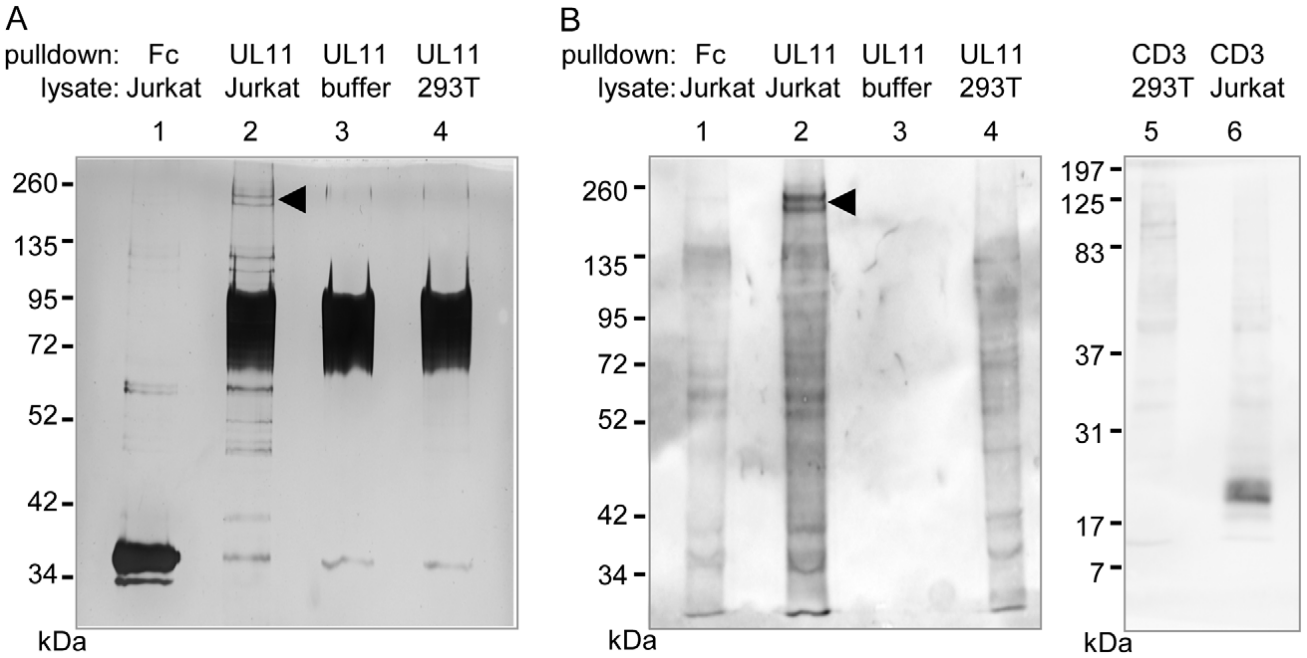
pUL11 interacts with a T cell surface protein with an approximate mass of 200 kDa. (A) Lysates of Jurkat or 293T cells, or lysis buffer, were incubated with UL11Fc (UL11) or the Fc control protein (Fc) and protein A sepharose beads. The bound proteins were separated by SDS-PAGE and detected by silver staining. (B) Jurkat or 293T cells were biotinylated prior to lysis. Proteins interacting with UL11Fc, the Fc domain or a CD3ε antibody were precipitated as in (A) and detected after blotting using HRP-streptavidin. A doublet at approx. 200 kDa is indicated.

To identify interaction partners of pUL11, proteins were precipitated from Jurkat cell lysates using UL11Fc as bait. A doublet of approximately 200 kDa and a few smaller proteins precipitated from Jurkat cell lysates by UL11Fc were detectable by silver staining (Figure 3A, lane 2). The 200 kDa bands were not present when the Fc control protein was used as bait (Figure 3A, lane 1) or when proteins were precipitated from 293T cells with UL11Fc (Figure 3A, lane 4).

Since pUL11 interacts with a leukocyte surface protein, we wished to determine which of the proteins precipitated from Jurkat lysates were surface proteins. Jurkat or 293T cells were labeled with membrane impermeable biotin before lysis and precipitation with UL11Fc, the Fc domain alone, or as a positive control with an antibody specific for the CD3 ε-chain. Following blotting and detection using peroxidase-coupled streptavidin (Figure 3B), the 200 kDa doublet produced the strongest signal of the proteins from Jurkat lysates precipitated by UL11Fc (Figure 3B, lane 2), whereas these proteins were not precipitated by the Fc domain, or the CD3-ε antibody (Figure 3B, lanes 1 and 6). As expected a 23 kDa protein immunoprecipitated by the CD3-ε antibody from Jurkat cell lysates could be visualized (Figure 3B, lane 6). These data suggest that the 200 kDa proteins are on the surface of Jurkat cells.

To determine the identity of these proteins, the double band was subjected to mass spectrometric analysis. Eight peptides stemming from the receptor tyrosine phosphatase CD45 were detected, and no other peptides corresponding to surface proteins, suggesting that CD45 is the interaction partner of pUL11.

### The interaction partner of pUL11 is the receptor tyrosine phosphatase CD45

**Figure 4:**
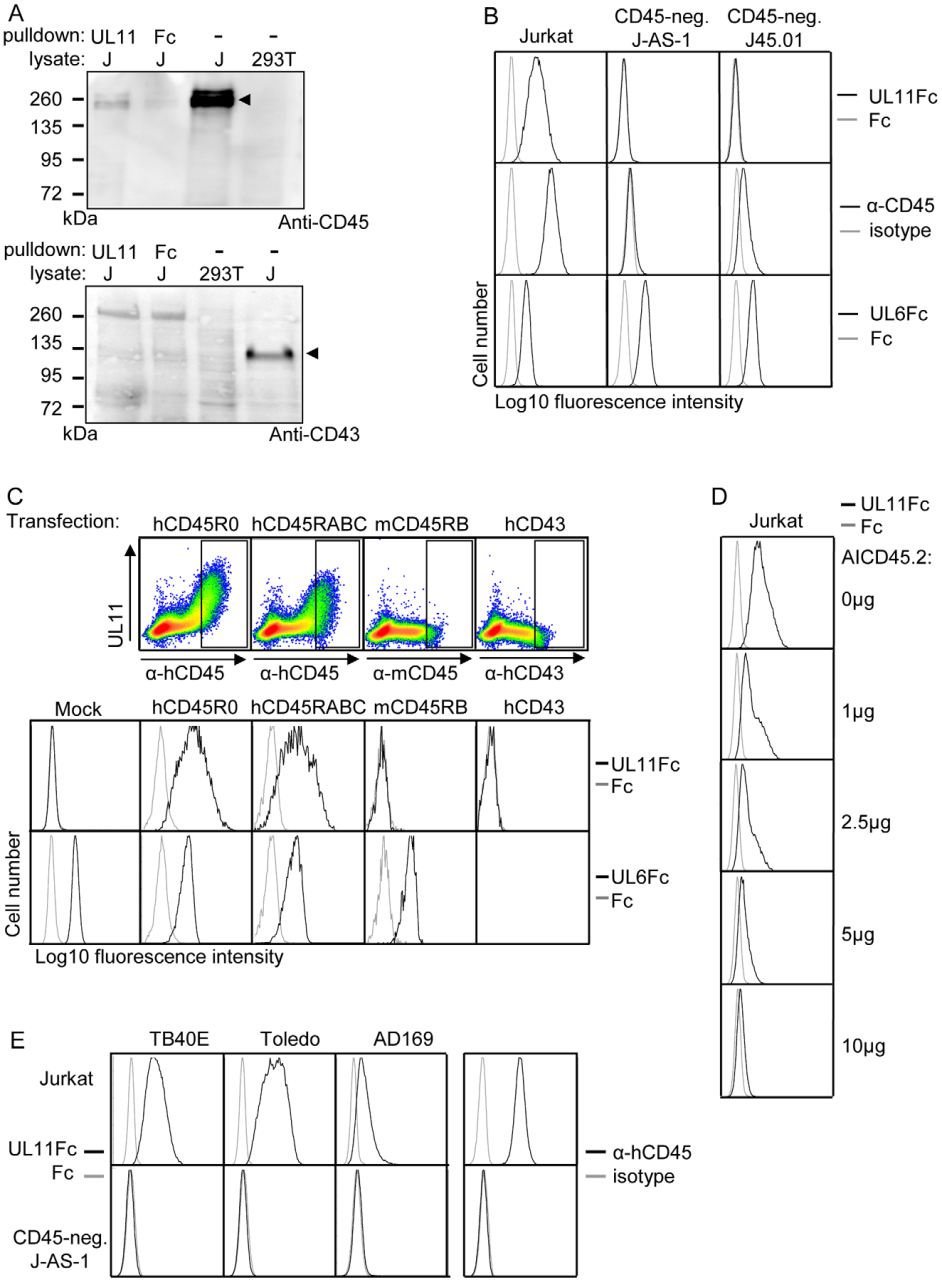
pUL11 interacts with CD45. (A) Immunoblot with anti-CD45 (upper panel) or anti-CD43 (lower panel) of lysates from 293T and Jurkat (J) cells and proteins precipitated from Jurkat cell lysates with UL11Fc or Fc control protein. Bands corresponding to CD45 and CD43 proteins in Jurkat lysates are indicated. (B) Flow cytometric analysis of the interactions of UL11Fc (black lines, top panel), Fc control protein (grey lines, top panel), CD45 antibody (black lines, middle panel) and UL6Fc (black lines, bottom panel) with Jurkat cells or the Jurkat derived cell lines J-AS-1 and J45.01. Grey lines in middle panels, staining with an isotype-matched control antibody. (C) Binding of UL11Fc (top row, density plots and second row, black lines), Fc control (grey lines) or UL6Fc (third row, black lines) to 293T cells that were mock transfected or transiently transfected with plasmids encoding either human CD45RABC or CD45R0 isoforms, mouse CD45RB or human CD43. Expression of hCD45, mCD45 and hCD43 is shown in density plots in upper panels with rectangles indicating gates for cells analyzed for UL11Fc and UL6Fc binding (rows 2 and 3). 48 h post transfection, cells were incubated with the indicated Fc proteins and co-stained with PE-labeled anti-IgG and FITC-labeled anti-human CD45 or anti-mouse CD45 or anti-human CD43 antibodies. (D) Inhibition of binding of UL11Fc to Jurkat cells by the AICD45.2 CD45 antibody. Cells were incubated with the indicated amounts of AICD45.2 for 30 min, prior to incubation with UL11Fc (black lines) or Fc control protein (grey lines). (E) Interactions of UL11Fc derived from TB40E, Toledo or AD169 strains of CMV (black lines, 3 leftmost columns), or Fc control protein (grey lines, 3 leftmost columns) with Jurkat (top row) or J-AS-1 cells (bottom row). Staining with anti-CD45 (black lines) or an isotype-matched control antibody (grey lines) is shown in the right hand column.

To confirm the interaction of pUL11 with CD45, the proteins precipitated from Jurkat cell lysates by UL11Fc were analyzed by immunoblotting with an antibody against CD45 (Figure 4A). CD45 was detectable in Jurkat, but not 293T cell lysates (Figure 4A, upper panel, lanes 3 and 4), and corresponding reactivity was observed with the 200 kDa protein doublet precipitated from Jurkat lysates by UL11Fc but not the control Fc domain (Figure 4A, upper panel, lanes 1 and 2). This confirms that pUL11 can interact with CD45 from Jurkat cell lysates. As a control for the specificity of the interaction, we also analyzed the precipitated proteins by immunoblotting using an antibody against CD43. CD43 has no homology to CD45, but a similarly sized and glycosylated ectodomain to CD45RABC [53], and the lectin MGL, which interacts with CD45, also binds to CD43 in Jurkat cells [54]. CD43 could be detected in Jurkat lysates (Figure 4A, lower panel, lane 4), but not in proteins precipitated by UL11Fc (Figure 4A, lower panel, lane 1).

CD45 is expressed on the surface of all nucleated hematopoietic cells [55] and could therefore be the interaction partner of pUL11 seen upon flow cytometric analysis of leukocytes (Figure 2B). To test this assumption, we analyzed the interaction of pUL11 with T cell lines that do not express CD45. The J-AS-1 cell line is a Jurkat cell line in which CD45 expression has been selectively disrupted by the stable expression of antisense RNA [56]. The J45.01 cell line was independently derived from Jurkat cells by irradiation and selection for loss of CD45 expression [57]. In both of these cell lines, the lack of CD45 expression coincided with the lack of UL11Fc binding (Figure 4B), indicating that CD45 expression is needed for the interaction. To show that CD45 expression is sufficient to induce the interaction of pUL11, we expressed the R0 and RABC isoforms of CD45 in 293T cells by transient transfection. In both cases, an interaction of UL11Fc with the cells expressing CD45 could be seen (Figure 4C). The amount of UL11Fc that binds to the cell surface increases with higher surface expression of CD45, indicating that the interaction is concentration dependent (Figure 4C, top row). Cells were also transiently transfected with expression plasmids encoding other surface glycoproteins; murine CD45 and human CD43. No interactions of UL11Fc were detected with cells expressing these proteins (Figure 4C, top and middle rows). Furthermore, binding of the extracellular domain of another member of the RL11 family, pUL6 (Figure S2), to 293T cells was not affected by transfection with either of the CD45 isoforms (Figure 4C, bottom row). The presence of CD45 on the cell surface therefore appears to be sufficient to induce a specific interaction with pUL11 and no detectable interactions occur between pUL11 and other T cell surface proteins.

To confirm by another method that pUL11 binds to Jurkat cells via CD45, we incubated Jurkat cells with antibodies directed against CD45 and then analyzed subsequent UL11Fc binding by flow cytometry. The AICD45.2 antibody that recognizes all isoforms of CD45 [58] blocked UL11Fc binding in a concentration dependent manner (Figure 4D). A second pan-CD45 antibody, MEM-28, had a marginal effect, and the UCHL-1 antibody that recognizes only the CD45R0 isoform [59] had no effect at all on UL11Fc binding (Figure S3). This experiment therefore supports the conclusion that pUL11 binding is CD45 dependent.

The UL11 protein shows a high degree of polymorphism between different strains of CMV [25], [29] and so, in order to determine whether the interaction with CD45 is restricted to pUL11 from the TB40/E strain of CMV, or a more general property of the protein, we investigated pUL11 from two additional CMV strains; Toledo and AD169. The predicted extracellular domains of the Toledo and AD169 UL11 proteins were also expressed as Fc fusion proteins and the binding of all three variants of UL11Fc to Jurkat and CD45 negative J-AS-1 cells was compared (Figure 4E). All three forms of pUL11 interacted with Jurkat but not with CD45-negative J-AS-1 cells, interestingly with some apparent quantitative differences in binding. This experiment indicated that the interaction of pUL11 with CD45 is not strain-specific.

**Figure 5:**
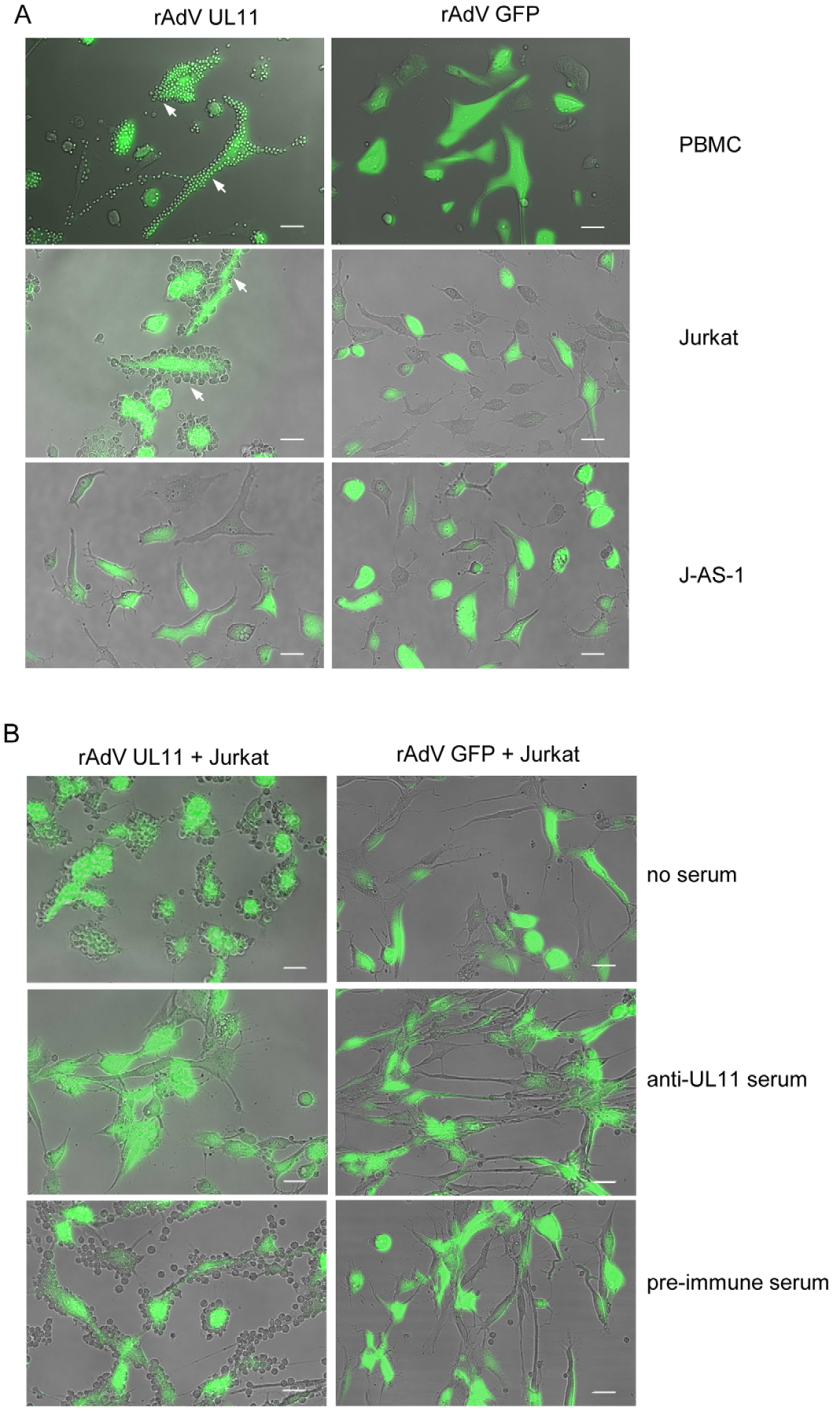
Surface expressed pUL11 mediates cell adhesion. (A) PBMCs, Jurkat or J-AS-1 cells were incubated with human fibroblasts that were transduced 3 days earlier with recombinant adenoviruses expressing pUL11 and GFP (rAdV UL11) or GFP alone (rAdV GFP). Unbound cells were removed by washing. White arrows indicate adhering cells, green cells show adenovirus derived GFP. Size bar, 50 µm. (B) Human fibroblasts transduced and depicted as in (A) were left untreated (top row), or incubated for 2 h with anti-UL11 (middle row) or pre-immune rabbit serum (bottom row) prior to incubation with Jurkat cells. Unbound cells were removed by washing. Size bar, 50 µm.

We were interested in whether the complete, surface expressed UL11 protein also interacts with CD45. To investigate this question, we transduced HFF cells with rAdV UL11 or the control rAdV GFP adenovirus and incubated these presenter cells with PBMCs, Jurkat or J-AS-1 cells lacking CD45. After washing away the unbound cells, rosetting of PBMCs and Jurkat cells around the pUL11 expressing cells could be clearly seen (Figure 5A, top two rows), and was absent from the control cells. No rosetting of the J-AS-1 cells was seen (Figure 5A, third row), indicating requirements for both CD45 and pUL11 for the interaction to take place. The binding of Jurkat cells to rAdV UL11 transduced fibroblasts could be disrupted following treatment of the transduced cells with UL11-specific antiserum, but not with pre-immune serum (Figure 5B, middle and bottom rows). This indicates that the adhesion properties of the soluble extracellular UL11Fc protein are also representative of full-length transmembrane pUL11.

### pUL11 interacts with CD45 on both naïve and activated T cells

**Figure 6:**
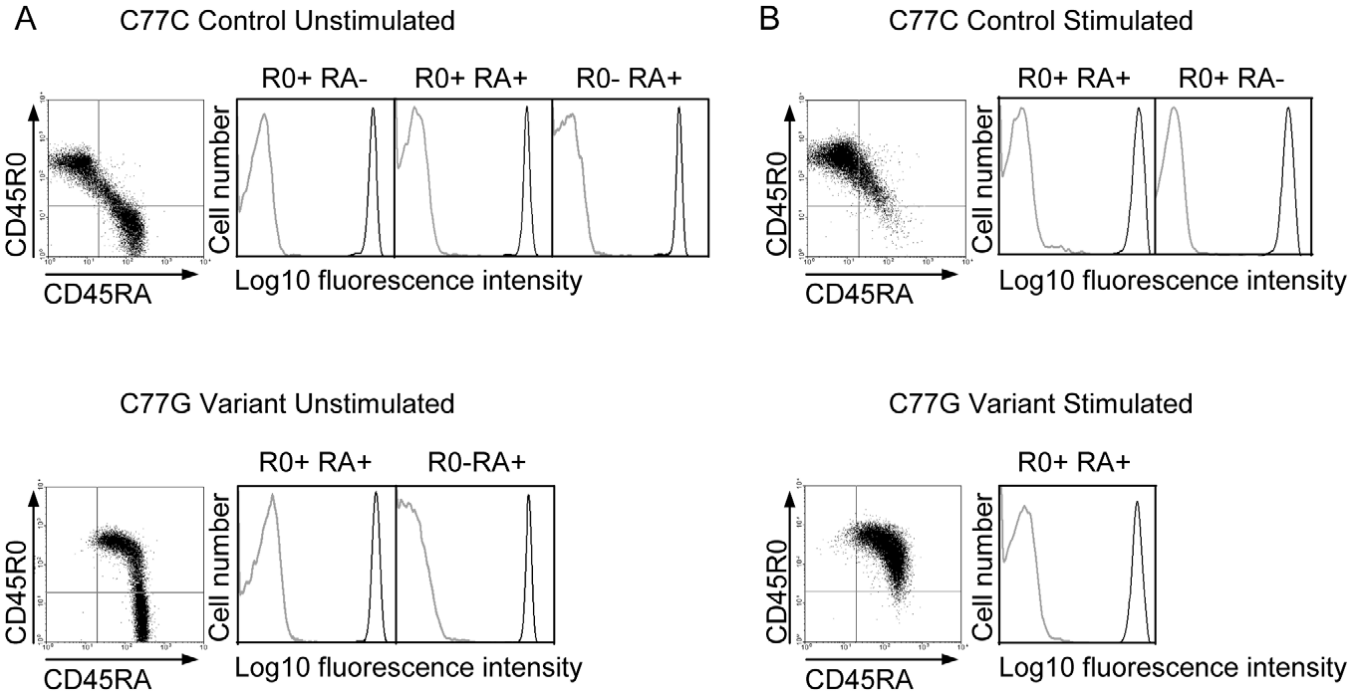
pUL11 interacts with T cells expressing both long and short isoforms of CD45. Primary T cells from donors with control or variant CD45 isoform expression were either left untreated (A) or stimulated with phytohaemagglutinin and interleukin-2 (B), and subsequently incubated with UL11Fc (black lines) or the Fc control protein (grey lines) and co-stained with anti-CD45RA or anti-CD45R0 antibodies. Interaction of the Fc proteins is depicted for cells gated as R0 positive, RA positive, or R0 and RA double positive as indicated.

Five different isoforms of CD45, generated by variation in splicing, have been detected in human lymphocytes. The expression of these isoforms is tightly controlled, depending on cell type, stimulation and maturation [60]. Naïve T cells typically express high molecular weight isoforms of CD45 containing exon A encoded domains. The RA isoforms are downregulated during activation. The expression of the R0 isoform, due to removal of exons A, B and C by splicing, is the major CD45 protein species characteristic for primed and memory T cells [61]. In individuals with a variant form of the CD45 gene, typified by the C77G polymorphism, the splicing pattern of CD45 is altered, meaning that T cells expressing both long RA and short R0 isoforms of CD45 are present after activation [62], [63]. In addition to CD45 splicing, the glycosylation of the different isoforms is also affected by cell stimulation, which could potentially affect the interaction with pUL11 [60]. It was of interest to understand whether pUL11 interacts preferentially with forms of CD45 associated with a particular activation state of T cells. Primary T cells from both control (C77C) and variant (C77G) individuals were therefore stained with antibodies against RA and R0 isoforms of CD45 and co-incubated with UL11Fc (Figure 6A). Binding of UL11Fc could be seen to cells expressing either RA, or R0 or both types of CD45 isoforms. Upon stimulation of the cells with phytohaemagglutinin and interleukin-2, the CD45 isoform expression pattern changed, but UL11Fc binding to all cell populations was unaltered (Figure 6B). This indicates that pUL11 can interact with both long and short isoforms of CD45 on both naïve and mature T cells.

### pUL11 disrupts T cell signaling and inhibits proliferation

**Figure 7:**
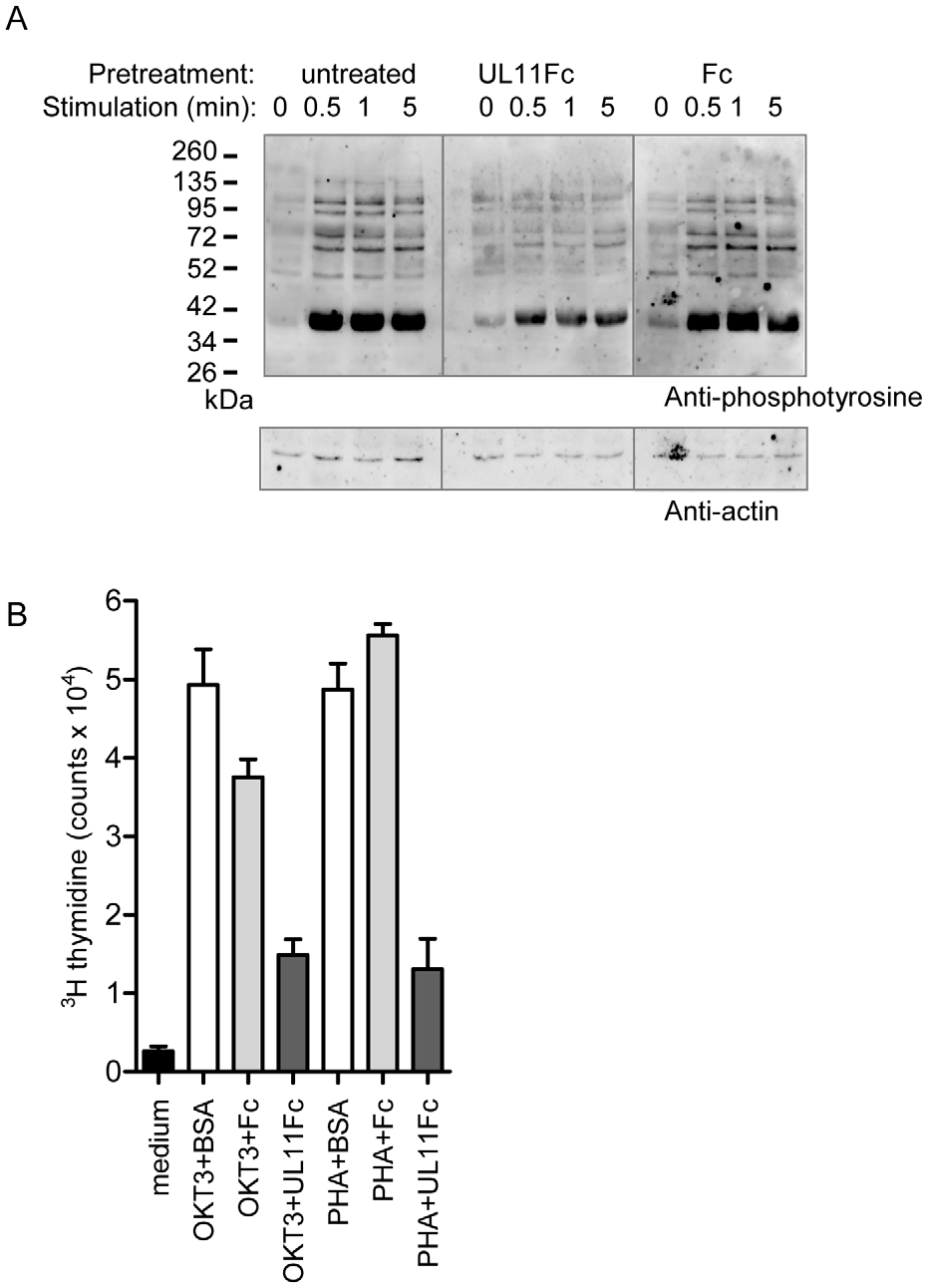
pUL11 treatment results in reduced T cell signaling and proliferation. (A) Immunoblot with the 4G10 phosphotyrosine-specific antibody (upper panel) or anti-actin (lower panel) of lysates from Jurkat cells that were either left untreated or were pretreated with UL11Fc or the Fc domain (2.5 µg) for 30 min prior to stimulation with a Jurkat TCR-specific antibody (C305) for the indicated times. (B) Primary T cells were either left untreated (medium) or were incubated with the CD3-specific antibody OKT3 or with phytohaemagglutinin (PHA) together with BSA, UL11Fc or the Fc domain (2 µg) for 3 days. Following incubation for 16 h with ^3^[H]-thymidine radionucleotide incorporation was measured. All samples were handled in triplicate. Representative data from one of three experiments are shown.

CD45 in T cells functions to prime the tyrosine kinase Lck, enabling TCR dependent signaling leading to activation and proliferation. Stimulation through the TCR-CD3 complex activates a signaling cascade resulting in the increased tyrosine phosphorylation of multiple downstream signaling intermediates [64]. To investigate the effect of pUL11 on this function of CD45, we stimulated Jurkat T cells with the C305 anti-Jurkat TCR antibody in the presence and absence of UL11Fc and detected induced changes in tyrosine phosphorylation by immunoblotting. In untreated cells and cells preincubated with the Fc control protein, the expected increase in tyrosine phosphorylation was readily detectable upon TCR stimulation (Figure 7A, left and right panels). In cells preincubated with UL11Fc, however, this increase was strongly reduced (Figure 7A, middle panel), indicating an inhibitory effect of pUL11 on T cell signaling.

Activation of T cells through TCR signaling leads to their proliferation. To determine whether T cell proliferation is also disrupted by pUL11 treatment, we measured the effects of UL11Fc on the proliferation of primary T cells in response to stimulation via CD3 (Figure 7B). T cells were incubated with the OKT3 CD3 antibody or with the mitogen phytohaemagglutinin, in the presence of UL11Fc or the Fc control protein. After 72 h, proliferation was measured and an inhibitory effect of UL11Fc could be seen. pUL11 therefore affects T cell functions that require active CD45, resulting in reduced TCR signaling and proliferation.

## Discussion

Immune suppression induced by acute CMV infection can have serious consequences for patients with impaired immune functions, such as an increased incidence of severe secondary bacterial and fungal infections in solid organ transplant recipients [65]. As a starting point to identify new CMV encoded immunosuppressive proteins, we considered the RL11 gene family. The RL11 proteins are largely uncharacterized, but the majority possesses the RL11 domain, a variable region of between 65 and 82 residues that has some sequence homology to the adenovirus CR1 domain and to immunoglobulin domains [26]. Adenovirus proteins containing the CR1 domain include immunomodulatory E3 proteins [26], [66], and immunoglobulin domains are commonly required for both cellular and viral protein interactions with cell surface components of the immune system [22], [23].

Acute CMV infection results in a reduction in the proliferation capacity of lymphocytes, which are not themselves infected by the virus [11]. A similar effect is produced *in vitro* upon contact between lymphocytes and CMV-infected cells, indicating the potential existence of uncharacterized surface expressed viral proteins with immunomodulatory properties [12], [21]. As it has previously been proposed that the RL11 domain containing protein pUL11 is expressed on the surface of human fibroblasts infected with the AD169 laboratory strain of CMV [29], we considered pUL11 to be a good candidate for a novel immunosuppressive protein. The surface staining of CMV-infected cells with a UL11-specific rabbit serum was interpreted by Hitomi et al. [29] as proof of the surface expression of UL11, however, it might rather have reflected the binding of rabbit immunoglobulins to the virally encoded Fc-receptors, as has previously reported by other authors [67], [68]. Despite the published description of pUL11 surface expression [29], we could only observe low levels of UL11 mRNAs in fibroblasts or epithelial cells infected with the AD169 or TB40/E strains of CMV (data not shown), in agreement with data using the Merlin strain of CMV (Andrew Davison, personal communication). Therefore, we first re-evaluated whether the UL11 protein from the TB40/E strain of CMV can be expressed on the surface of fibroblasts and epithelial cells, using a recombinant adenovirus expression system. The surface expression of pUL11 that we detected then led us to search for interactions between pUL11 and surface molecules on different cell types. The predicted extracellular domain of pUL11 was used in flow cytometry binding studies and interacted with leukocyte cell lines and primary leukocytes, but not with control cell lines of non-hematopoietic origin, indicating an interaction with a leukocyte specific receptor. Mass spectrometry analysis of surface proteins pulled down by the pUL11 extracellular domain from Jurkat cell lysates identified the receptor tyrosine phosphatase CD45 as a binding partner of pUL11. That CD45 is also responsible for the interaction of pUL11 with leukocytes seen in flow cytometry analysis was confirmed using two different CD45 deficient cell lines. No interactions could be seen between pUL11 and the Jurkat derived CD45 negative cell lines J-AS-1 or J45.01 [56], [57]. The interaction of pUL11 with the surface of leukocytes could also be demonstrated using fibroblasts expressing full-length pUL11, to which PBMCs and CD45 expressing T cells adhered. The interaction of pUL11 with Jurkat cells could be blocked by pretreatment with either an CD45 antibody, or pUL11 antiserum, confirming the specificity of the interaction. As pUL11 shows sequence variation between different strains of CMV [25], [29], we purified the predicted extracellular domain of pUL11 from two additional CMV strains, Toledo and AD169, and showed that these forms of pUL11 also interact specifically with CD45 expressing cell lines, apparently with some differences in affinities, demonstrating that the interaction is not a peculiarity of the TB40/E form of pUL11. Expression of two different isoforms of CD45 in 293T cells both induced pUL11 binding, indicating that CD45 is sufficient for the interaction. A second member of the RL11 family, pUL6, was used to investigate whether the interaction with CD45 is a general property of RL11 proteins, or specific to pUL11. No changes in pUL6 binding were seen in relation to CD45 expression, indicating that the interaction is a particular property of pUL11. An interaction with pUL11 could not be induced by expression of the mouse CD45 protein, or the human CD43 glycoprotein in 293T cells. In conjunction with the observation that pUL11 binding is abrogated in the CD45 deficient T cell lines, this provides strong evidence that pUL11 interacts with CD45 and that the interaction is specific.

CD45 exists as a set of different isoforms, the expression and glycosylation of which is tightly controlled and depends on cell type and maturation state. We demonstrated that the interaction of pUL11 with primary T cells is not dependent upon T cell activation state, as interactions were detectable between pUL11 and both long and short isoforms of CD45, expressed on both naïve and mature T cells. This implies that the immunomodulatory effects of pUL11 *in vivo* may be wide ranging, potentially affecting both priming and effector functions of T cells.

The interaction of pUL11 with CD45 is markedly different from that of other known CD45 ligands. The other CD45 ligands that have been described are all lectins, which recognize oligosaccharide moieties with specificities determined by the lectin carbohydrate recognition domains [69]. Lectins typically bind to multiple ligands and have pronounced differences in their interactions with the various CD45 isoforms and glycoforms due to their differing glycosylation patterns [60]. The C-type lectin macrophage galactose type lectin (MGL), a pattern recognition receptor on myeloid antigen presenting cells which recognizes N-acetylgalactosamine (GalNAc) sugars, for example, binds only to the longer isoforms of CD45 due to their higher GalNAc content, and also to the sialoglycoprotein CD43 [54]. Other lectins are even more specific in their preferences; glucosidase II and serum-mannan binding protein only interact with CD45 glycoforms characteristically found on immature thymocytes; in the case of serum-mannan binding protein only with the hybrid-type N-linked glycans on the R0 isoform [70], [71]. Lectin ligands for CD45 frequently do not show reduced surface binding to CD45 negative T cell lines, due to the abundance of other suitably glycosylated ligands [54], [72], and in contrast to the binding pattern observed for pUL11. As pUL11 interacts with diverse forms of CD45 and shows no detectable binding to CD45 negative T cells, its interaction with CD45 seems to be of a different nature from those of previously described ligands.

CD45 is necessary for T cell functions mediated via signaling through the TCR complex. The Src family kinase Lck is primed by CD45-mediated dephosphorylation of tyrosine 505, which releases an intramolecular bond holding Lck in a closed, inactive conformation [50]. In the absence of primed Lck, signal transduction through the TCR cannot be initiated [73] and inhibition of TCR mediated signaling functions is therefore characteristic of reduced CD45 function [74]. Pretreatment with pUL11 reduced the cascade of tyrosine phosphorylation triggered by T cell stimulation with an anti-TCR antibody and TCR dependent T cell proliferation was also inhibited. These effects indicate that signal transduction through the TCR is impaired in the presence of pUL11, and are consistent with a restriction in CD45 function.

The critical control of signaling thresholds by CD45 implies that its effects must be tightly regulated. Although this process is not yet fully understood, mechanisms have been proposed, some of which are influenced by interactions of ligands with the extracellular domain of CD45, and might therefore be applicable to pUL11 function. For CD45 to function correctly, it must have a tightly controlled localization in the plasma membrane, with regulated contact to substrate proteins. Lck is present in lipid rafts [75], and a fraction of CD45 must have access to Lck to be able to dephosphorylate residue Y505 and generate a pool of primed Lck. This partial localization of CD45 in lipid rafts is dependent on the extracellular domain of CD45 and may require interactions in *cis* with other raft components [53], [76]. As CD45 is a potent phosphatase, it is however important that only a fraction is present in lipid rafts. Excessive contact between CD45 and the TCR signaling complex can result in dephosphorylation of the active site tyrosine 394 of Lck and potentially of Lck substrates such as ZAP-70 and the ζ-chain of the CD3-TCR complex, blocking the initiation of signal transduction [48], [77], [78]. CD45 is therefore excluded from the signaling complex by lipid raft movements [76], [79]. The inhibitory effects on CD45 function of the therapeutic anti-CD45RB mAb 6G3 [80], and also the lectin placental protein 14 [81] are associated with excess movement of CD45 into lipid rafts, allowing a deactivation of Lck. It is conceivable that an interaction with pUL11 could disrupt *cis* interactions of the extracellular domain of CD45 with lipid raft components, affecting the controlled partitioning of CD45 into lipid rafts and thus generating the observed effects on T cell signal transduction.

CD45 activity has also been described to be dependent on dimerisation state; a model has been proposed in which dimerisation results in the formation of an inhibitory structural “wedge” disrupting substrate access to the phosphatase domain [82], [83]. Reduced dimerisation associated with a prevalence of high molecular weight forms of CD45 has been suggested to underlie excessive CD45 activity resulting in hyperresponsive T cell function [84], [85]. Increased dimerisation, as seen by forced dimerisation of a EGFR-CD45 hybrid molecule and the interaction of the lectins galectin-1 and placental protein 14 with CD45 decreases CD45 function [81], [86], [87]. An analogous role for pUL11 is possible, in which pUL11 binding to the extracellular domain of CD45 increases dimerisation, decreasing CD45 phosphatase activity and therefore restricting TCR signaling.

Other mechanistic interpretations, such as allosteric regulation of CD45 phosphatase function upon ligand binding, potentially in conjunction with those suggested here, are of course also possible. Further investigations into the mechanisms of pUL11 function seem likely to lead to new insights into CD45 regulation.

The role of pUL11 in the context of CMV infection is intriguing; a transient general suppression of T cell function during viral infection has been demonstrated [9]–[13] and the interaction of pUL11 with CD45 may contribute to this effect. It is also clear that a means for the virus to escape from CMV-specific T cell control could enhance viral replication. The consequences of the interaction with CD45 may also extend beyond effects on T cell function as it is well known that CD45 plays important roles in other classes of leukocytes [50]. The observation that UL11 proteins from three different strains of CMV all bind to CD45, but apparently with some variation in affinity, is also interesting, as it may point towards strain or host dependent immunosuppressive effects of CMV infection. Before these questions can be addressed, however, the expression profile of pUL11 during CMV infection needs to be understood. We would speculate that the expression of pUL11 may be cell type or state specific, but this remains to be demonstrated.

In conclusion, we have identified CMV pUL11 as a novel, specific interaction partner of CD45. pUL11 limits T cell signaling and proliferation, effects which are consistent with a reduction in CD45 activity. The interaction of pUL11 with CD45 appears to represent a previously unknown pathway by which CMV can induce immunosuppression, with potential therapeutic significance.

## Materials and Methods

### Ethics statement

Human blood cells were provided by voluntary blood donors in the Institute of Transfusion Medicine, Hannover Medical School. All materials and data were analyzed anonymously. The use of the human blood cells was approved by the ethics committee of Hannover Medical School.

### Cells

Human lung adenocarcinoma epithelial A549 cells and human foreskin fibroblasts (HFF) were propagated in DMEM containing 10% FCS, 2 mM glutamine and 1% non-essential amino acids. 293T and 293A cells were maintained in DMEM containing 10% FCS. Jurkat T cells, J45.01 cells [57] and J-AS-1 cells [56], were cultured in RPMI 1640 with 2 mM glutamine and 10% FCS, with the medium for the latter two cell lines supplemented with 20 mM HEPES and for the J-AS-1 cells, also with G418 (0.5 mg/ml). For protein production, retinal pigment epithelium (RPE) or 293T cells were maintained in serum free Pro293a-CDM (LONZA), containing 2 mM glutamine. PBMCs were flushed from leukocyte filters used to prepare erythrocytes from healthy voluntary blood donors for transfusion. Where indicated, the individuals were identified as carrying wild type or C77G variant CD45. PBMCs were isolated by density gradient centrifugation using Biocoll Separating Solution or Ficoll (both from Biochrom) and cryopreserved until usage. PBMCs were maintained in RPMI 1640 containing 20 mM HEPES or 1 mM sodium pyruvate, 4 mM glutamine and 10% FCS.

### Recombinant adenoviruses

The recombinant adenoviruses rAdV UL11, rAdV GFP and rAdV UL6Fc are based on the pAdZ-CV5 replication deficient adenovirus vector [51]. First, the sequence for the V5 epitope tag (GKPIPNPLLGLDST) was added at the 3′-end of the UL11 open reading frame (ORF) in the CMV TB40/E genome [52] by homologous recombination in *E. coli* as previously described [88]. The UL11V5 fragment was amplified using the primers 5′-AGT­CGG­ATC­CAA­TTACCTGTGGTAGAATGC-3′ and 5′-GGC­CGG­ATC­CTT­ACG­TAG­AAT­CAA­GAC­C­TA-3′ and cloned into the pIRES eGFP vector (BD Biosciences Clontech). The UL11V5 IRES eGFP cassette was then introduced into the AdZ-CV5 vector by homologous recombination in the *E.coli* SW102 strain as previously described [51]. rAdV GFP, rAdV UL6Fc, rAdV Toledo UL11Fc and rAdV AD169 UL11Fc were constructed by introducing the ORFs for GFP, Toledo UL11Fc, AD169 UL11Fc and for UL6Fc (see below) into the pAdZ-CV5 vector. The correct construction of the adenovirus genomes was confirmed by restriction analysis and sequencing. Recombinant adenoviruses were produced and titered in 293A cells.

### Fc fusion protein and antiserum production

The sequence encoding the predicted extracellular domain of pUL11 was amplified from the genome of the CMV strain TB40/E [52] using the primers 5′-CGGGATCCATCA­GCC­T­C­C­ACGATGCCTG-3′ and 5′-CCGGTCGACTGTAGCCACGTGTTGGTGC-3′ and cloned into a pCR3-based vector containing sequences encoding the mouse IgH signal peptide and the Fc region of human IgG1 [89]. The predicted extracellular domains of pUL11 from CMV strains Toledo and AD169 were amplified from the respective viral genomes [90], [91] using the following primers and cloned into the same vector. pUL11 Toledo: 5′-CGGGATCCATCAGCCTCCATGATGCCTG-3′ and 5′-CCGGTCGACTGT­GGC­CAC­G­T­G­TTGGTGC-3′. pUL11 AD169: 5′-CGGGATCC­A­TCA­GTTTCCACGACCATGC-3′ and 5′-CCGGTCGACTGTCGCCACGTGTTGGTAC-3′. The sequence encoding the predicted extracellular domain of pUL6 was amplified with the following primers: 5′-CGG­GAT­CCC­A­T­G­CTAAGATAAACGGGTGG-3′ and 5′-CCG­GTC­GAC­GAA­TGC­CAA­GTTA­GTTA­TGT­T­C-3′ and cloned in an analogous manner. The ORF for the TB40/E UL11Fc protein was amplified with the primers 5′-CGG­CGG­CCG­CGC­CAC­CAT­GAA­CTTCGGGTTC-3′ and 5′-CGGAATTCTCATTTACCCGGAGACAGGG-3′ and cloned into the pSFbeta91-wpre replication deficient retrovirus vector [92]. The ORF for the Fc domain of human IgG1 was cloned into the pSFbeta91-wpre vector in a similar manner. Retroviruses were generated by transfecting the Phoenix-gp packaging cell line with the pSFbeta91-wpre constructs together with the retroviral gag/pol plasmid M25-DAW [93] and the feline endogenous retrovirus envelope glycoprotein expression plasmid RD114 [94] and used to transduce 293T cells as described [92]. TB40/E UL11Fc, Toledo UL11Fc, AD169 UL11Fc, UL6Fc and Fc control proteins were purified from serum free supernatants of retrovirally transduced 293T cells or adenovirally transduced RPE cells by protein A affinity chromatography using hiTrap rProtein A FF columns (GE Healthcare, Munich, Germany). The TB40/E UL11Fc protein was used to generate a rabbit antiserum directed against the extracellular domain of pUL11 (Pineda Antikoerper Service, Berlin, Germany).

### Plasmid construction and transfection

Plasmids LCA.1 and LCA.6 that express the human CD45R0 and CD45RABC isoforms under control of the SR-alpha promoter [56] were kindly provided by David Rothstein. The CD45R0 ORF was removed from LCA.1 by treatment with *Eco RI* and *Sal I* and replaced by an *Mfe I/Sal I* treated PCR fragment encoding the transmembrane region and cytoplasmic tail of mouse CD45, which was amplified with primers 5′-GGCCAATT­GACGCGT­GCGGCC­G­C­T­A­T­A­TT­C­C­T­G­GTGTTTCTGA-3′ and 5′-GGCCAATT­GCCCG­TCG­A­CCG­T­T­A­TGAA­C­T­C­T­GGGTT­G­G­A­GCTG-3′, using a plasmid carrying the mouse CD45RB cDNA [95]. The resulting plasmid was cut with *Not I* and a PCR fragment was added encoding the extracellular domain of mouse CD45RB, which was amplified from the mouse CD45RB cDNA using primers 5′-GGCGCGGCCGC­ACG­CGT­A­G­­G­­G­GCACAGCTGAT­C­T­C­­C­A­G­­AT-3′ and 5′-GGCGCGGCCGCTTT­AGCATTA­AAA­T­T­T­­GTTGACTCATTTC-3′, leading to the mCD45 expression vector. A PCR fragment encoding the extracellular domain of human CD43 was generated with primers 5′-CCC­GCG­G­CC­GCTGTT­TCTTAG­GGACA­C­G­­GC-3′ and 5′-GAG­GCG­GCC­GCG­CCT­CGT­GAG­TTC­T­C­A­TCTGGGTTCC-3′ from a cDNA vector encoding human CD43 (Open Biosystems), and was cloned in an analogous manner, resulting in the CD43 expression vector.

1×10^6^ HEK293T cells were transfected with 4 µg of the expression constructs using the Lipofectamine 2000 reagent, and flow cytometric analysis as described below was performed 48 h later. Detection of protein expression from the transfected cells was using FITC-coupled MEM-28 anti-human CD45 (Immunotools), FITC-coupled MEM-59 anti-human CD43 antibody (Immunotools) or FITC-coupled IBL-5/25 anti-mouse CD45 (Immunotools).

### Flow cytometry and microscopy

Cell surface expression of pUL11 was measured in HFF or A549 cells, 72 h after transduction with the rAdV at a multiplicity of infection (MOI) of 500 and 300, respectively. The pUL11-specific antiserum was adsorbed for 8 h on uninfected A549 or HFF cells before use. Cells were incubated with antiserum in blocking solution (1% BSA, 0.1% gelatine, 2 mM EDTA in PBS) followed by PE-conjugated goat anti-rabbit antibody (Open Biosystems) in blocking solution containing 6% goat serum. All steps were performed at 4°C. For flow cytometry based binding assays, 2.5 µg of purified Fc fusion proteins were incubated with 1×10^6^ cells in blocking solution (5% mouse serum, 2 mM EDTA in PBS). Bound Fc proteins were detected using PE-conjugated anti-human IgG (Acris). To determine the effects of CD45 antibodies on Fc protein binding, cells were incubated with MEM-28 (Immunotools), UCHL-1 [59] (kindly provided by P. Beverley, University of Oxford, UK) or AICD45.2 [58] (kindly provided by B. Schraven, University of Magdeburg, Germany) antibodies in blocking solution for 30 min prior to incubation with UL11Fc fusion protein. Sub-populations of PBMCs were identified using antibodies directed to the following surface markers; T cells: anti-CD3-FITC (Immunotools), anti-CD4-Dy647 (Acris), anti-CD8-PE-Dy590 (Antibodies-online). B cells: anti-CD19-PE-Dy590 (Antibodies-online). NK cells: anti-CD56-APC (Immunotools); NK cells were identified as CD56 positive and CD3 negative cells, monocytes: anti-CD14-APC (Immunotools) and neutrophils: anti-CD15-FITC (BD). Measurements were performed on a Beckmann Coulter Cytomics FC500 cytometer and analyzed using CXP analysis software. FACS based binding assays to stimulated CD4 T cells were performed using CD4 T cells prepared from PBMCs from control or variant (CD45 C77G) donors by positive MACS separation (Miltenyi Biotec) using the OKT-4 anti-CD4 mAb purified from hybridoma. The purity of the CD4 positive fraction was determined by FACS. Cells were stained and measured immediately or were stimulated with 1 µg/ml PHA (Murex Diagnostics Ltd.) for 24 h and then treated for 8 days with 25 U/ml IL-2 (Roche). For staining, the cells were incubated in 50% mouse serum in PBS, followed by Fc fusion protein (1 µg) for 45 min. After washing, cells were incubated with FITC-conjugated anti-CD45RA and APC-conjugated anti-CD45R0 antibodies (BD). Bound Fc fusion proteins were detected using PE-conjugated anti-human IgG (Acris). Measurement was performed using a FACSCalibur cytometer and analysis was performed using WinMDI software, version 2.9. For confocal microscopy A549 or HFF cells infected with rAdVs as described above were incubated with the anti-pUL11 serum in blocking solution (1% BSA, 0.1% gelatine, PBS), followed by Alexa 568 conjugated goat anti-rabbit (Invitrogen). Cells were fixed with 3% paraformaldehyde and observed using a Zeiss LSM 510 Meta Confocal Microscope. To observe leukocyte rosetting, HFF infected 96 h earlier with rAdVs at an MOI of 500 were co-cultured with Jurkat T cells, J-AS-1 T cells or freshly isolated PBMCs at a ratio of 1∶20 for 2 h at 37°C, and washed 8 times with PBS. To determine the effects of antisera on leukocyte rosetting, HFF cells infected with rAdVs as described were incubated for 2 h at 37°C with 400 µl of the rabbit anti-UL11 serum or preimmune serum diluted with 600 µl of DMEM. The serum was then removed and the HFF co-cultured with E6.1 Jurkat T cells (2×10^6^ Jurkat cells per 1×10^5^ HFF) for another 2.5 h, followed by 15 washing steps with PBS. Images were taken using a Zeiss Axio Observer light/epifluorescence microscope.

### Protein analysis

Glycosylation was investigated using purified Fc proteins or proteins of lysates from A549 cells transduced with rAdV at an MOI of 100 72 h earlier and prepared using NP40 lysis buffer (150 mM NaCl, 1% NP40, 10 mM Tris-HCl pH 7.4, 1 mM EDTA, protease inhibitor cocktail [Calbiochem]). Cell lysates or purified proteins were boiled for 5 min in denaturing buffer (0.5% SDS, 0.5% 2-mercaptoethanol) before being treated with N-glycosidase F (4 U) (Roche) or Endo-α-N-acetylgalactosaminidase (2,000 U) and neuraminidase (100 U) (New England Biolabs) in 500 mM sodium phosphate buffer pH 7.6 containing 1% NP40 for 2 h or overnight at 37°C. Cell surface proteins were biotinylated by incubating 2.5×10^7^cells/ml in PBS with 2 mM Sulfo-NHS-LC-Biotin (Thermo Fisher Scientific), for 30 min. The cells were washed three times with 100 mM glycine in PBS and then lysed in NP-40 lysis buffer. Proteins were pulled down or immunoprecipitated from cell lysates prepared from 1×10^8^ cells/ml of NP40 lysis buffer. 500 µl of cell lysate precleared by incubation for 20 min with protein A sepharose CL-4B (GE Healthcare) was incubated with 10 µg of Fc protein or antibody and 20 µl protein A sepharose CL-4B for 90 min at 4°C. CD3 was immunoprecipitated using OKT3 (eBioscience). The preparative pull-down for mass spectrometric analysis used 2×10^8^ cells lysed in 1 ml of NP40 lysis buffer, incubated with 20 µg of protein and 20 µl of protein A sepharose CL-4B. For silver staining of proteins, SDS-PAGE gels were washed twice in 50% methanol/10% acetic acid for 15 min each, once in 10% ethanol/5% acetic acid for 6 min, and rinsed twice for 9 min in water. Gels were then incubated in sodium hydrosulfite (20 ng/ml) for 9 min, followed by 0.1% silver nitrate solution, containing 0.75 µl/ml 37% formaldehyde for a further 9 min. Gels were then rinsed for 30 s in water and transferred to a 3% sodium carbonate solution containing 0.1% of 37% formaldehyde and 10 ng/ml sodium thiosulfate. Development was halted using stop solution (2.5% acetic acid, 5% Tris). For detection of pUL11, CD45, CD43 and phosphotyrosine proteins by immunoblotting a mouse anti-V5 antibody (Invitrogen), the rabbit anti-pUL11 serum, the MEM-28 CD45 antibody (Immunotools), the MEM-59 CD43 antibody (Immunotools) and the 4G10 phosphotyrosine antibody (Millipore) were used, respectively, followed by incubation with the appropriate HRP-conjugated anti-mouse or anti-rabbit antibodies (Dako).

### T cell assays

Induction of tyrosine phosphorylation was measured after the incubation of Fc fusion proteins (2.5 µg) with 4×10^5^ Jurkat cells in 100 µl of culture medium for 30 minutes at 37°C, followed by stimulation with 100 µl of C305 anti-Jurkat TCR mAb [96] hybridoma supernatant (kindly provided by B. Schraven, University of Magdeburg, Germany). Stimulation was stopped by the addition of 1 ml ice-cold PBS and the cell suspension was immediately centrifuged. The cell pellet was then lysed with NP-40-digitonin lysis buffer (1% NP-40, 1% digitonin, 50 mM Tris-HCl pH 7.4, 150 mM NaCl, 10 mM EDTA, 2 mM sodium vanadate and protease inhibitor cocktail [Calbiochem]). To measure proliferation of PBMCs, Fc fusion proteins (2.5 µg) and OKT3 (1 µg; purified from hybridoma supernatant) were adsorbed onto Maxi-Sorb 96-well plates. 1×10^5^ PBMCs per well were incubated in 200 µl of culture medium. PHA (Oxoid, Basingstoke, UK) was added where indicated at 25 µg/ml. After 48 h, 0.4 µCi [^3^H]-thymidine (Amersham Biosciences, Braunschweig, Germany) was added. After 24 h the cells were harvested and incorporated [^3^H]-thymidine measured in a beta-counter (Perkin Elmer, Rodgau, Germany).

### Mass spectrometric analysis

Protein bands were excised manually from a preparative, Coomassie-stained gel. After destaining two times with 100 µl of 50% acetonitrile (ACN), 20 mM NH_4_HCO_3_ at 37°C for 30 min, bands were dehydrated by adding 100 µl ACN and dried. 20 µl of sequencing grade trypsin (10 ng/ml; Promega) was added and after 30 min incubation on ice remaining trypsin solution was discarded. Digestion was continued at 37°C overnight and stopped by adding 0.1% trifluoroacetic acid, 50% ACN. Tryptic peptides were extracted with two times 20 µl 50% ACN, 0.1% formic acid (FA) for 30 min at 37°C and 10 µl ACN for 30 min at RT. All extracts were combined and dried in a vacuum centrifuge.

For LC-iontrap-MS analysis peptide samples were dissolved in 10 µl 10% ACN. Five microliter per peptide sample were injected onto a C18 RP-Column (Zorbax SB, C18, 80 Å, 5 µm, 150×0,5 mm: Agilent) using a 1100 Series Agilent HPLC System equipped with an autosampler, coupled online to an Esquire3000**^+^** ion trap mass spectrometer (Bruker Daltonics). Using a two buffer system (A: 5% ACN, 0,1% FA; B: 80% ACN, 0,1% FA) and a flow rate of 5 µl/min, a multi-step gradient was applied after injection: 0–5 min: 0% B; 30 min gradient to 53.9% B (40% ACN); 5 min gradient to 100% B; increase of flow rate to 10 µl/min in 1 min; 10 min at 100% B; 4 min gradient to 0% B; 15 min at 0% B. The MS method used to select and fragment the eluting peptides was set to trigger fragmentation of the three most intensive peaks from an MS scan at a 40,000 ion count threshold and a preference of doubly charged ions. Automated precursor exclusion after one acquired spectrum per precursor for 0.3 min was used. The ESI source conditions were set to 10 psi nebulizer gas pressure with dry gas heated to 300°C at a flow rate of 4.0 l/min. Mass spectrometrical data were searched against the SwissProt Database with carbamidomethylation of cysteins as static and oxidation of methionines as variable modification. For ion trap-MS 150 ppm mass deviation was tolerated for precursors and 0.7 Da for peptide fragments in MS/MS. At least two peptides with a Mascot peptide ion score higher than 25 each were used as a threshold for protein identification.

## Supporting Information

Figure S1 **Specificity of the rabbit antiserum raised against the predicted extracellular domain of pUL11.** Immunoblot with the rabbit antiserum raised against the fusion protein consisting of the predicted extracellular domain of pUL11 and the human IgG Fc domain (c.f. Figure S2). The rabbit serum was pre-absorbed to rAdV GFP transduced cells to reduce non-specific interactions. Lysates of A549 cells transduced with rAdV UL11 (UL11), rAdV GFP (GFP), or left uninfected (U) were used to prepare immunoblots and proteins were detected using pUL11 anti-serum or for comparison using an antibody specific for the V5 epitope. Bands corresponding to the major 50 kDa form (arrow) and to high molecular weight forms of pUL11 (bracket) are indicated.
(TIF)

Figure S2 **Generation of the pUL11 and pUL6 Fc fusion proteins.** (A) Cartoons of the domains of the Fc fusion proteins. (B, C) The UL11Fc and UL6Fc proteins harvested from the supernatants of transduced or transfected 293T cells and purified using protein A sepharose were separated by SDS-PAGE and detected by Coomassie blue staining or by immunoblotting using HRP conjugated anti-human IgG. (D) The UL11Fc or the control Fc protein were treated with PNGase F and detected after immunoblotting as in (C).
(TIF)

Figure S3 **Blocking effects of CD45 antibodies on the interaction of pUL11 with Jurkat cells.** Jurkat cells were left untreated (top panel) or incubated with 10 µg of the indicated CD45 antibodies for 30 min, prior to incubation with UL11Fc (black lines) or the Fc control protein (grey lines).
(TIF)
