# Supplementary figures and images for: The Human Cytomegalovirus UL11 Protein Interacts with the Receptor Tyrosine Phosphatase CD45, Resulting in Functional Paralysis of T Cells
Source: PLoS Pathog. 2011 Dec 8;7(12):e1002432. doi: 10.1371/journal.ppat.1002432 (PMC3234252; doi:10.1371/journal.ppat.1002432)

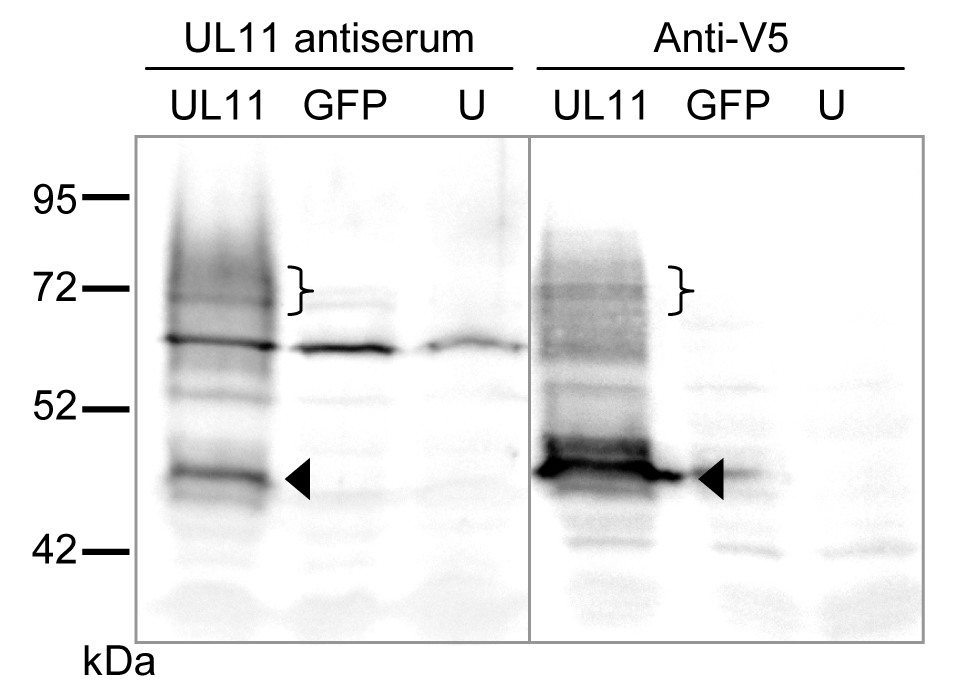

Supplement: Figure S1 — Specificity of the rabbit antiserum raised against the predicted extracellular domain of pUL11. Immunoblot with the rabbit antiserum raised against the fusion protein consisting of the predicted extracellular domain of pUL11 and the human IgG Fc domain (c.f. Figure S2). The rabbit serum was pre-absorbed to rAdV GFP transduced cells to reduce non-specific interactions. Lysates of A549 cells transduced with rAdV UL11 (UL11), rAdV GFP (GFP), or left uninfected (U) were used to prepare immunoblots and proteins were detected using pUL11 anti-serum or for comparison using an antibody specific for the V5 epitope. Bands corresponding to the major 50 kDa form (arrow) and to high molecular weight forms of pUL11 (bracket) are indicated. (TIF) [file ppat.1002432.s001.tif]

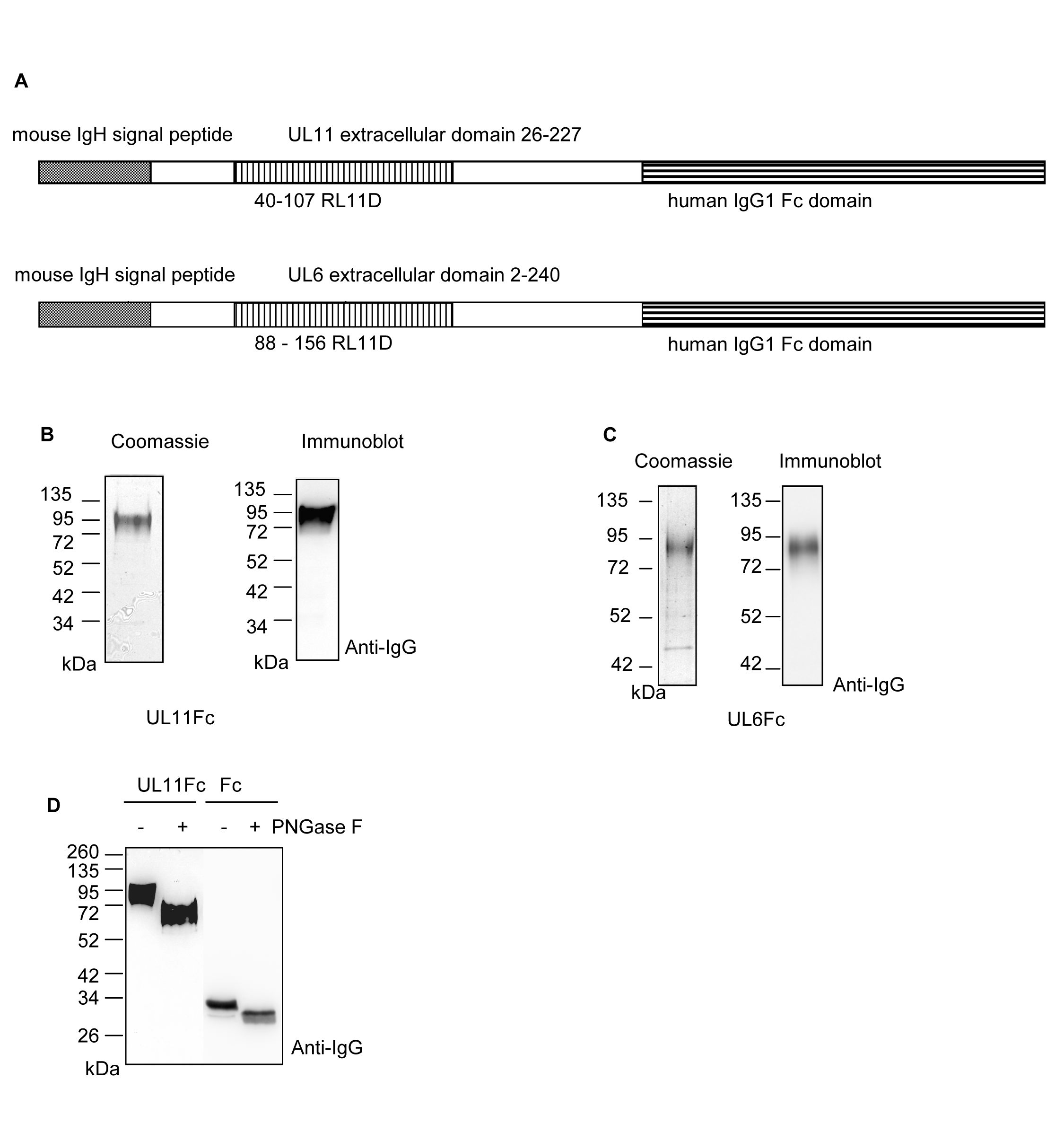

Supplement: Figure S2 — Generation of the pUL11 and pUL6 Fc fusion proteins. (A) Cartoons of the domains of the Fc fusion proteins. (B, C) The UL11Fc and UL6Fc proteins harvested from the supernatants of transduced or transfected 293T cells and purified using protein A sepharose were separated by SDS-PAGE and detected by Coomassie blue staining or by immunoblotting using HRP conjugated anti-human IgG. (D) The UL11Fc or the control Fc protein were treated with PNGase F and detected after immunoblotting as in (C). (TIF) [file ppat.1002432.s002.tif]

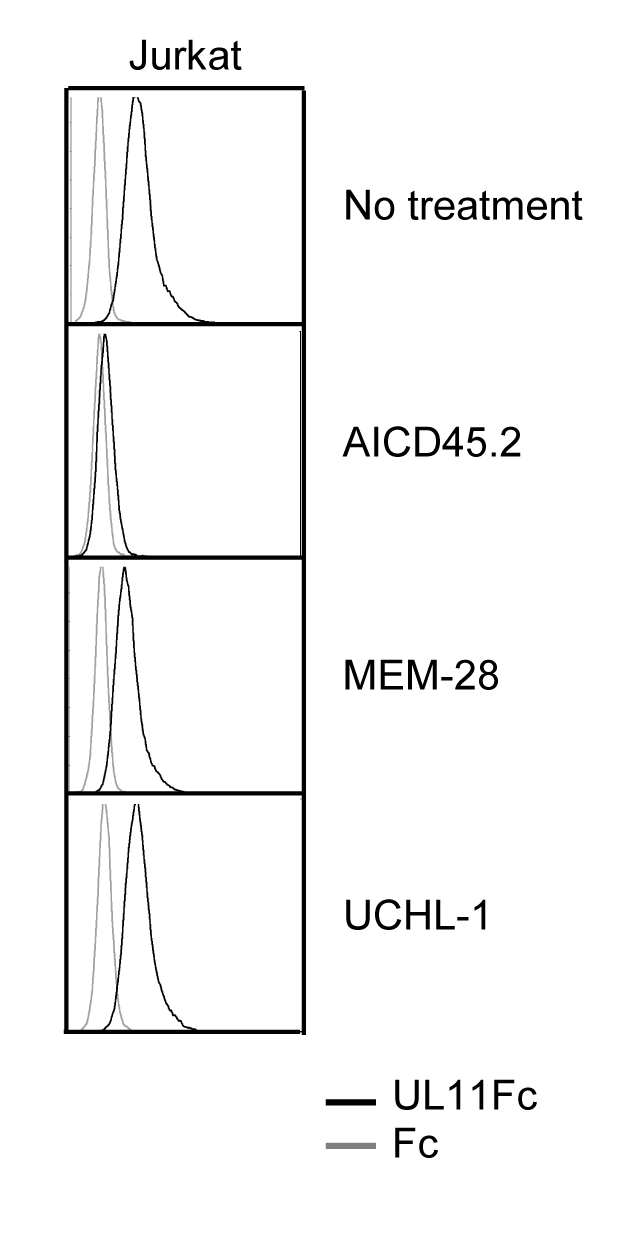

Supplement: Figure S3 — Blocking effects of CD45 antibodies on the interaction of pUL11 with Jurkat cells. Jurkat cells were left untreated (top panel) or incubated with 10 µg of the indicated CD45 antibodies for 30 min, prior to incubation with UL11Fc (black lines) or the Fc control protein (grey lines). (TIF) [file ppat.1002432.s003.tif]
